# Supplementary figures and images for: Factors driving the compositional diversity of Apis mellifera bee venom from a Corymbia calophylla (marri) ecosystem, Southwestern Australia
Source: PLoS One. 2021 Jun 30;16(6):e0253838. doi: 10.1371/journal.pone.0253838 (PMC8244862; doi:10.1371/journal.pone.0253838)

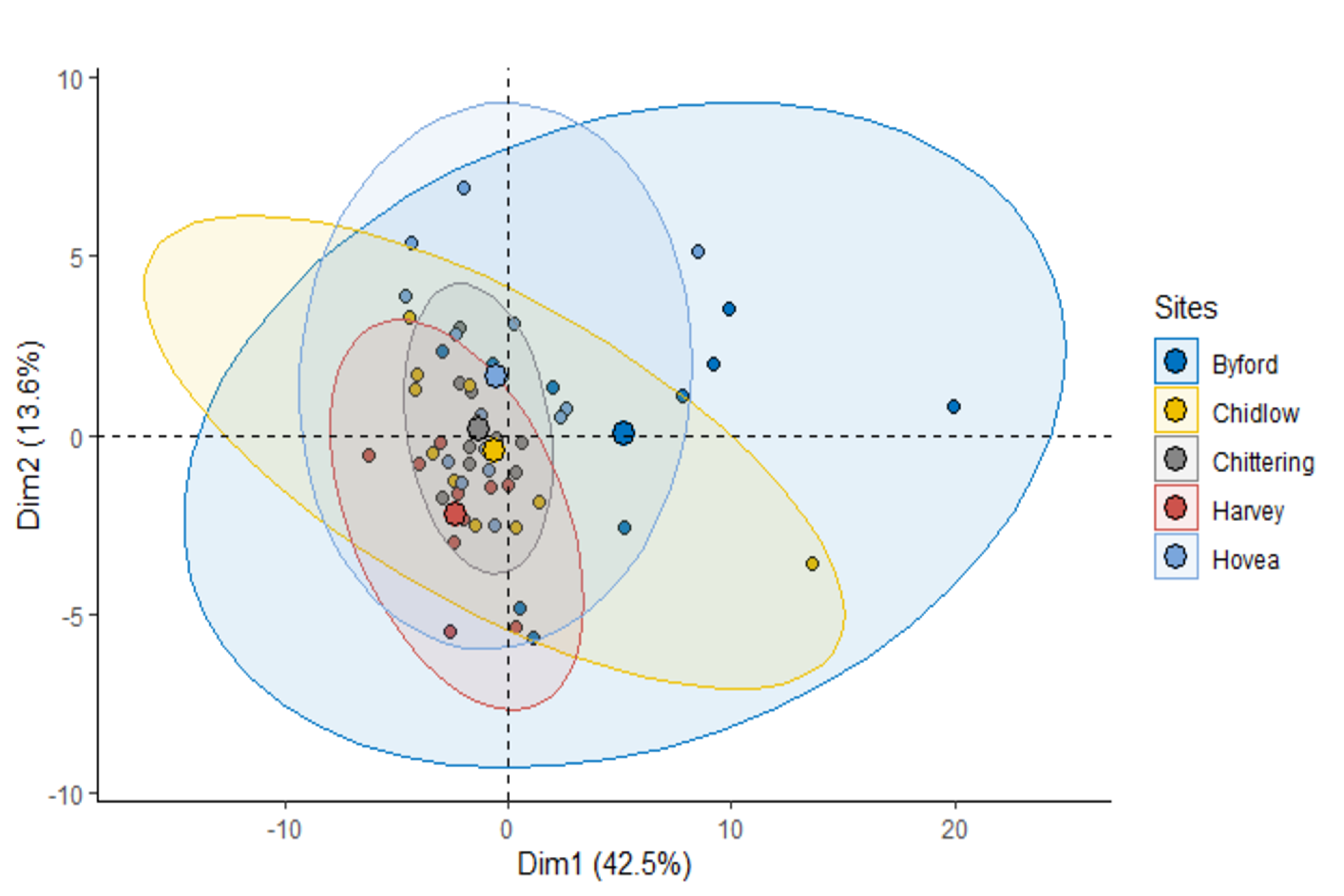

Supplement: S1 Fig — Biplot of the first and second axes obtained from the principal component analysis (95% confidence ellipses are shown) showing dependencies of protein profile between sampling sites. Points represent venom samples (n = 55). (TIFF) [file pone.0253838.s001.tiff]

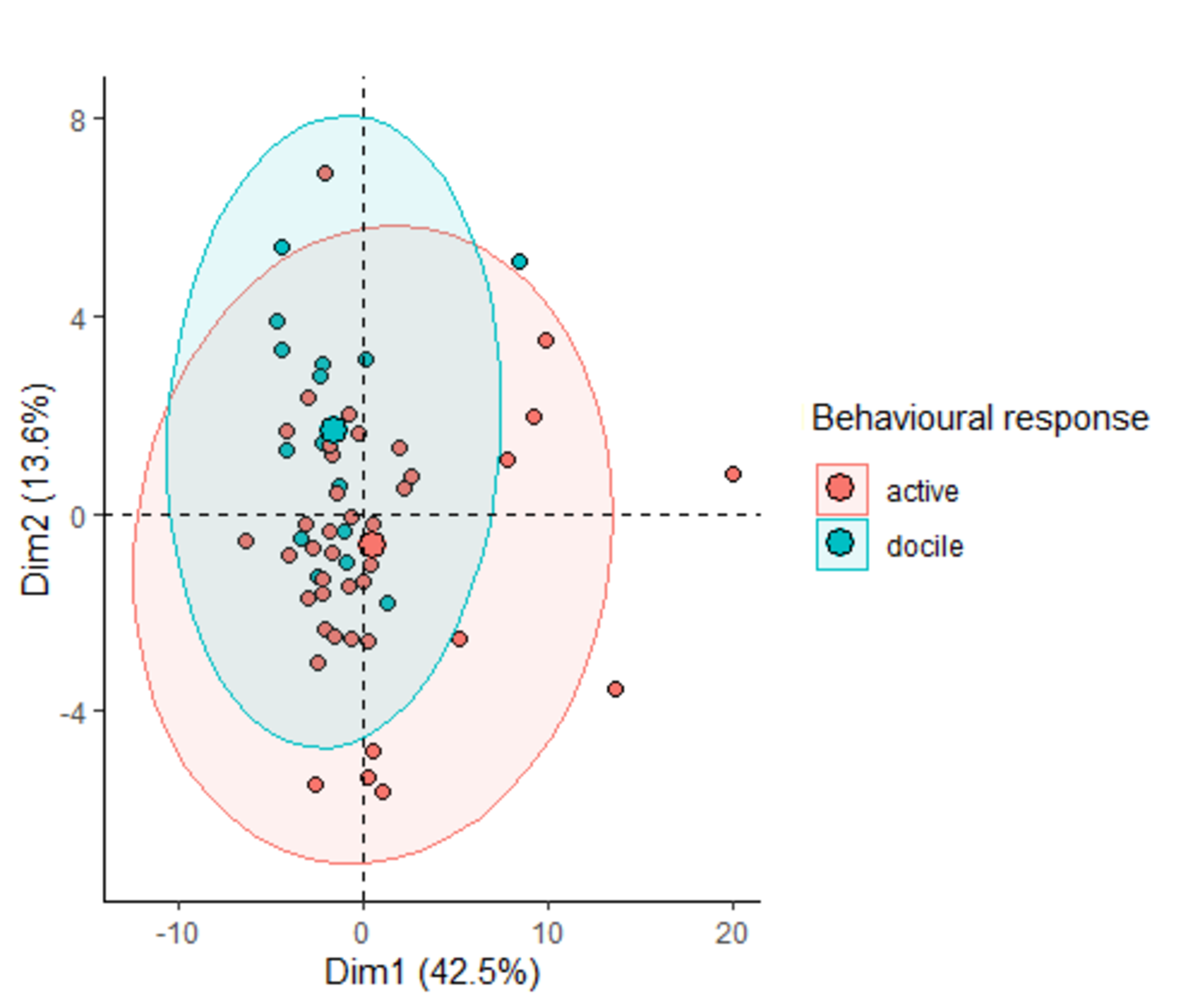

Supplement: S2 Fig — Biplot of the first and second axes obtained from the principal component analysis (95% confidence ellipses are shown) showing dependencies between active and docile bees. Points represent venom samples (n = 55). (TIFF) [file pone.0253838.s002.tiff]
