# Supplementary material for: Factors driving the compositional diversity of Apis mellifera bee venom from a Corymbia calophylla (marri) ecosystem, Southwestern Australia
Source: PLoS One. 2021 Jun 30;16(6):e0253838. doi: 10.1371/journal.pone.0253838 (PMC8244862; doi:10.1371/journal.pone.0253838)
Supplement: S1 Table — Pollen percent occurrence was calculated by scanning 500 or more pollen grains per sample. CC (ChemCentre), marri (Corymbia calophylla), Jarrah (Eucalyptus marginata), Blackbutt (Eucalyptus patens), Myrt. (Myrtaceae). (DOCX) [file pone.0253838.s003.docx]

**S1 Table.**

| **CC Number** | **Site** | **Marri** | **Jarrah** | **Blackbutt** | **Myrt. sp.** | **Other sp.** |
| --- | --- | --- | --- | --- | --- | --- |
| **A. Honey %** | | | | | | |
| 19S1990/169 | Harvey | 84.27 | 5.63 | 5.63 | 4.47 | 0.00 |
| 19S1990/170 | Harvey | 94.54 | 0.00 | 3.39 | 1.69 | 0.38 |
| 19S1990/171 | Harvey | 92.34 | 1.18 | 4.13 | 2.16 | 0.20 |
| 19S1990/172 | Byford | 85.96 | 2.92 | 5.46 | 4.87 | 0.78 |
| 19S1990/173 | Byford | 67.70 | 5.45 | 21.01 | 3.50 | 2.33 |
| 19S1990/174 | Byford | 76.63 | 6.70 | 9.96 | 2.11 | 4.60 |
| 19S1990/175 | Chittering | 85.44 | 7.38 | 1.36 | 4.85 | 0.97 |
| 19S1990/176 | Chittering | 83.51 | 7.10 | 1.88 | 6.05 | 1.46 |
| 19S1990/177 | Chittering | 76.92 | 7.50 | 4.81 | 9.04 | 1.73 |
| 19S1990/178 | Chidlow | 76.94 | 4.91 | 8.13 | 4.16 | 5.86 |
| **B. Pollen %** | | | | | | |
| 19S1992/062 | Harvey | 94.00 | 0.20 | 1.80 | 4.00 | - |
| 19S1992/063 | Byford | 87.40 | 1.77 | 0.00 | 5.31 | 5.51 |
| 19S1992/064 | Chittering | 74.23 | 9.20 | 9.36 | 6.44 | 0.77 |
