# Supplementary material for: Factors driving the compositional diversity of Apis mellifera bee venom from a Corymbia calophylla (marri) ecosystem, Southwestern Australia
Source: PLoS One. 2021 Jun 30;16(6):e0253838. doi: 10.1371/journal.pone.0253838 (PMC8244862; doi:10.1371/journal.pone.0253838)
Supplement: S2 Table — Protein identification was confirmed by tryptic peptide match to the Apis mellifera reference proteome, requiring at least two unique peptides. (✔) Proteins previously reported in literature accounts of bee venom proteomics (Li et al., 2013; Matysiak et al, 2014; Matysiak et al, 2016). MW, Molecular weight of intact protein; kDa (Kilodalton). (DOCX) [file pone.0253838.s004.docx]

**S2 Table.**

| **Protein**  **(**✔**previously reported)** |  | **UniProt Accession** | **Protein description** | **MW (kDa)** | **Coverage [%]** | **Peptides** | **PSMs** | **Unique Peptides** |
| --- | --- | --- | --- | --- | --- | --- | --- | --- |
| Phospholipase A2 | ✔ | P00630 | Phospholipase A2 OS=Apis mellifera OX=7460 PE=1 SV=3 | 19 | 86 | 63 | 498 | 12 |
| Melittin-N | ✔ | P0DPR9 | Melittin-N OS=Apis cerana OX=7461 GN=MELT PE=1 SV=1 | 7.6 | 70 | 24 | 119 | 4 |
| Melittin | ✔ | P01501 | Melittin OS=Apis mellifera OX=7460 GN=MELT PE=1 SV=1 | 7.6 | 70 | 40 | 336 | 20 |
| Venom acid phosphatase Acph-1 | ✔ | A0A087ZRC7 | Venom acid phosphatase Acph-1 OS=Apis mellifera OX=7460 GN=Acph-1 PE=3 SV=1 | 46.8 | 60 | 48 | 124 | 48 |
| Peptidyl-prolyl cis-trans isomerase | ✔ | A0A088A5R9 | Peptidyl-prolyl cis-trans isomerase OS=Apis mellifera OX=7460 PE=3 SV=1 | 20.1 | 59 | 11 | 40 | 11 |
| Apidaecins type 14 | ✔ | Q06601 | Apidaecins type 14 OS=Apis mellifera OX=7460 GN=APID14 PE=1 SV=1 | 19.4 | 58 | 3 | 18 | 3 |
| Hyaluronidase | ✔ | A0A088AMF4 | Hyaluronidase OS=Apis mellifera OX=7460 PE=3 SV=1 | 44.2 | 57 | 36 | 113 | 36 |
| PDGF_2 domain-containing protein |  | A0A088AUL3 | PDGF_2 domain-containing protein OS=Apis mellifera OX=7460 GN=Pvf1 PE=3 SV=1 | 35.9 | 57 | 23 | 71 | 23 |
| OBP14 | ✔ | Q1W640 | OBP14 OS=Apis mellifera OX=7460 PE=1 SV=1 | 15.2 | 54 | 6 | 8 | 6 |
| Apamin | ✔ | P01500 | Apamin OS=Apis mellifera OX=7460 PE=1 SV=2 | 5.2 | 52 | 11 | 54 | 11 |
| Major royal jelly protein 1 | ✔ | O18330 | Major royal jelly protein 1 OS=Apis mellifera OX=7460 GN=MRJP1 PE=1 SV=1 | 48.9 | 52 | 23 | 52 | 22 |
| Allergen Api m 6.03 / Api m 6.04 | ✔ | P83563 | Allergen Api m 6.03 / Api m 6.04 OS=Apis mellifera OX=7460 PE=1 SV=2 | 9.8 | 51 | 21 | 119 | 21 |
| Mast cell degranulating peptide | ✔ | A0A087ZPM2 | Mast cell degranulating peptide OS=Apis mellifera OX=7460 GN=Mcdp PE=4 SV=1 | 5.8 | 50 | 10 | 126 | 10 |
| Uncharacterized protein |  | H9K869 | Uncharacterized protein OS=Apis mellifera OX=7460 PE=4 SV=1 | 11.4 | 49 | 8 | 24 | 8 |
| Uncharacterized protein |  | A0A088AUP4 | Uncharacterized protein OS=Apis mellifera OX=7460 GN=LOC724341 PE=4 SV=1 | 27.5 | 48 | 14 | 27 | 14 |
| TIL domain-containing protein |  | A0A088A2Y0 | TIL domain-containing protein OS=Apis mellifera OX=7460 GN=100576444 PE=4 SV=1 | 9 | 48 | 3 | 11 | 3 |
| Uncharacterized protein |  | A0A088AL69 | Uncharacterized protein OS=Apis mellifera OX=7460 PE=4 SV=1 | 16.4 | 46 | 6 | 30 | 6 |
| Uncharacterized protein |  | A0A088AG18 | Uncharacterized protein OS=Apis mellifera OX=7460 GN=Sap-r PE=4 SV=1 | 100 | 44 | 40 | 99 | 40 |
| Chymotrypsin inhibitor | ✔ | A0A088AF77 | Chymotrypsin inhibitor OS=Apis mellifera OX=7460 GN=Amci PE=4 SV=1 | 8.1 | 43 | 2 | 3 | 2 |
| Icarapin variant 2 (Fragment) | ✔ | A0A096XH35 | Icarapin variant 2 (Fragment) OS=Apis mellifera carnica OX=88217 GN=icarapin PE=2 SV=1 | 22.4 | 43 | 18 | 98 | 2 |
| Uncharacterized protein |  | A0A087ZQ29 | Uncharacterized protein OS=Apis mellifera OX=7460 PE=4 SV=1 | 61.4 | 42 | 21 | 53 | 21 |
| Sep15_SelM domain-containing protein |  | A0A087ZTN5 | Sep15_SelM domain-containing protein OS=Apis mellifera OX=7460 GN=LOC410663 PE=4 SV=1 | 17.8 | 41 | 6 | 14 | 6 |
| Uncharacterized protein |  | A0A087ZN65 | Uncharacterized protein OS=Apis mellifera OX=7460 PE=3 SV=1 | 17.9 | 41 | 6 | 21 | 6 |
| Protein disulfide-isomerase |  | A0A088A7L2 | Protein disulfide-isomerase OS=Apis mellifera OX=7460 PE=3 SV=1 | 56.2 | 40 | 18 | 29 | 18 |
| Major royal jelly protein 9 | ✔ | Q4ZJX1 | Major royal jelly protein 9 OS=Apis mellifera OX=7460 GN=MRJP9 PE=2 SV=1 | 48.7 | 39 | 17 | 36 | 17 |
| Omega-conotoxin-like protein 1 |  | H9KQJ7 | Omega-conotoxin-like protein 1 OS=Apis mellifera OX=7460 PE=2 SV=1 | 8.3 | 38 | 3 | 9 | 3 |
| Glyco_18 domain-containing protein |  | A0A088AMK2 | Glyco_18 domain-containing protein OS=Apis mellifera OX=7460 PE=3 SV=1 | 50.1 | 36 | 11 | 17 | 11 |
| C1q-like venom protein | ✔ | A0A087ZXG5 | C1q-like venom protein OS=Apis mellifera OX=7460 PE=4 SV=1 | 18.7 | 36 | 6 | 14 | 6 |
| Tertiapin | ✔ | A0A087ZPM1 | Tertiapin OS=Apis mellifera OX=7460 PE=4 SV=1 | 6.1 | 36 | 2 | 24 | 2 |
| Uncharacterized protein |  | A0A088ACF4 | Uncharacterized protein OS=Apis mellifera OX=7460 PE=3 SV=1 | 72.4 | 35 | 20 | 40 | 17 |
| PA2c domain-containing protein | ✔ | A0A087ZZK8 | PA2c domain-containing protein OS=Apis mellifera OX=7460 GN=LOC724436 PE=3 SV=1 | 20.5 | 35 | 10 | 38 | 10 |
| Venom serine protease 34 | ✔ | Q8MQS8 | Venom serine protease 34 OS=Apis mellifera OX=7460 PE=2 SV=1 | 45.5 | 32 | 12 | 32 | 12 |
| Uncharacterized protein |  | A0A088A8Z2 | Uncharacterized protein OS=Apis mellifera OX=7460 GN=scf PE=4 SV=1 | 37.7 | 31 | 8 | 15 | 8 |
| Venom dipeptidyl peptidase 4 | ✔ | A0A088A1D0 | Venom dipeptidyl peptidase 4 OS=Apis mellifera OX=7460 PE=3 SV=1 | 88.4 | 31 | 26 | 56 | 26 |
| J domain-containing protein |  | A0A088AJE9 | J domain-containing protein OS=Apis mellifera OX=7460 PE=4 SV=1 | 40.4 | 30 | 10 | 32 | 10 |
| Gal_mutarotas_2 domain-containing protein |  | A0A088A7S9 | Gal_mutarotas_2 domain-containing protein OS=Apis mellifera OX=7460 GN=LOC551205 PE=3 SV=1 | 107 | 29 | 23 | 35 | 23 |
| Major royal jelly protein 8 | ✔ | Q6TGR0 | Major royal jelly protein 8 OS=Apis mellifera OX=7460 GN=Mrjp8 PE=2 SV=1 | 46.9 | 29 | 13 | 20 | 11 |
| Uncharacterized protein |  | A0A087ZYN8 | Uncharacterized protein OS=Apis mellifera OX=7460 PE=4 SV=1 | 11.2 | 28 | 6 | 35 | 6 |
| Peptidase S1 domain-containing protein |  | A0A088ADM5 | Peptidase S1 domain-containing protein OS=Apis mellifera OX=7460 GN=SP3 PE=3 SV=1 | 39.5 | 26 | 7 | 14 | 7 |
| Uncharacterized protein |  | A0A087ZYW8 | Uncharacterized protein OS=Apis mellifera OX=7460 PE=4 SV=1 | 34.3 | 25 | 9 | 13 | 9 |
| Uncharacterized protein |  | A0A088A6Z2 | Uncharacterized protein OS=Apis mellifera OX=7460 GN=Lys-3 PE=4 SV=1 | 17.1 | 25 | 2 | 2 | 2 |
| Glutathione peroxidase |  | A0A088AB47 | Glutathione peroxidase OS=Apis mellifera OX=7460 GN=Gtpx2 PE=3 SV=1 | 23.1 | 24 | 4 | 5 | 4 |
| Venom carboxylesterase-6 | ✔ | B2D0J5 | Venom carboxylesterase-6 OS=Apis mellifera OX=7460 PE=2 SV=1 | 63.6 | 24 | 12 | 33 | 12 |
| Secapin | ✔ | A0A088AL68 | Secapin OS=Apis mellifera OX=7460 GN=LOC406145 PE=4 SV=1 | 8.7 | 23 | 3 | 98 | 3 |
| Secapin | ✔ | I1VC85 | Secapin OS=Apis mellifera OX=7460 PE=2 SV=1 | 8.8 | 23 | 3 | 53 | 3 |
| Uncharacterized protein |  | A0A088A015 | Uncharacterized protein OS=Apis mellifera OX=7460 GN=LOC408851 PE=3 SV=1 | 100 | 23 | 19 | 37 | 19 |
| Protein disulfide-isomerase |  | A0A088A7Y2 | Protein disulfide-isomerase OS=Apis mellifera OX=7460 GN=ERp60 PE=3 SV=1 | 55.8 | 22 | 9 | 12 | 9 |
| Uncharacterized protein |  | A0A087ZP00 | Uncharacterized protein OS=Apis mellifera OX=7460 GN=Crc PE=3 SV=1 | 47.1 | 22 | 9 | 11 | 9 |
| Thioredoxin domain-containing protein |  | A0A088AA01 | Thioredoxin domain-containing protein OS=Apis mellifera OX=7460 GN=Trx1-like3 PE=4 SV=1 | 49.3 | 22 | 7 | 9 | 7 |
| Uncharacterized protein |  | A0A087ZU97 | Uncharacterized protein OS=Apis mellifera OX=7460 GN=100578816 PE=4 SV=1 | 10.9 | 22 | 3 | 4 | 3 |
| Uncharacterized protein |  | A0A087ZTZ3 | Uncharacterized protein OS=Apis mellifera OX=7460 GN=100577847 PE=4 SV=1 | 42.2 | 21 | 7 | 11 | 7 |
| alpha-1,2-Mannosidase |  | A0A088AW08 | alpha-1,2-Mannosidase OS=Apis mellifera OX=7460 PE=3 SV=1 | 43.7 | 20 | 7 | 14 | 7 |
| Uncharacterized protein |  | A0A088AP79 | Uncharacterized protein OS=Apis mellifera OX=7460 GN=LOC409790 PE=4 SV=1 | 24.3 | 20 | 4 | 9 | 4 |
| Peptidase M12B domain-containing protein |  | A0A087ZQA1 | Peptidase M12B domain-containing protein OS=Apis mellifera OX=7460 GN=LOC409468 PE=4 SV=1 | 86.3 | 20 | 11 | 14 | 11 |
| Uncharacterized protein |  | A0A088A722 | Uncharacterized protein OS=Apis mellifera OX=7460 PE=4 SV=1 | 48.4 | 20 | 6 | 10 | 6 |
| Chitin-binding type-2 domain-containing protein | ✔ | A0A088APL1 | Chitin-binding type-2 domain-containing protein OS=Apis mellifera OX=7460 GN=Cht5 PE=3 SV=1 | 69 | 19 | 9 | 18 | 9 |
| Apolipophorin-III-like protein | ✔ | B0LUE8 | Apolipophorin-III-like protein OS=Apis mellifera OX=7460 GN=A4 PE=2 SV=1 | 21.3 | 19 | 2 | 2 | 2 |
| EF-hand domain-containing protein |  | A0A088AUN3 | EF-hand domain-containing protein OS=Apis mellifera OX=7460 GN=LOC552747 PE=4 SV=1 | 59.8 | 19 | 6 | 6 | 6 |
| Uncharacterized protein |  | A0A087ZRB6 | Uncharacterized protein OS=Apis mellifera OX=7460 GN=LOC725215 PE=3 SV=1 | 42.6 | 18 | 5 | 12 | 5 |
| Uncharacterized protein |  | A0A087ZWI6 | Uncharacterized protein OS=Apis mellifera OX=7460 GN=LOC408327 PE=4 SV=1 | 36.1 | 18 | 4 | 7 | 4 |
| Uncharacterized protein |  | A0A087ZYR0 | Uncharacterized protein OS=Apis mellifera OX=7460 PE=4 SV=1 | 55 | 17 | 5 | 8 | 5 |
| Gb3_synth domain-containing protein |  | A0A088A691 | Gb3_synth domain-containing protein OS=Apis mellifera OX=7460 GN=727510 PE=4 SV=1 | 40.8 | 17 | 5 | 7 | 5 |
| Peptidase S1 domain-containing protein |  | A0A088AEY5 | Peptidase S1 domain-containing protein OS=Apis mellifera OX=7460 PE=3 SV=1 | 29.5 | 17 | 4 | 12 | 4 |
| Major royal jelly protein 2 | ✔ | O77061 | Major royal jelly protein 2 OS=Apis mellifera OX=7460 GN=MRJP2 PE=1 SV=1 | 51 | 16 | 6 | 9 | 5 |
| Uncharacterized protein |  | A0A087ZR24 | Uncharacterized protein OS=Apis mellifera OX=7460 PE=4 SV=1 | 33.2 | 16 | 3 | 5 | 3 |
| Uncharacterized protein |  | A0A087ZSL8 | Uncharacterized protein OS=Apis mellifera OX=7460 PE=4 SV=1 | 32.2 | 16 | 4 | 6 | 4 |
| Uncharacterized protein |  | A0A087ZPM0 | Uncharacterized protein OS=Apis mellifera OX=7460 PE=4 SV=1 | 58.4 | 15 | 6 | 8 | 6 |
| Uncharacterized protein |  | A0A088ARP1 | Uncharacterized protein OS=Apis mellifera OX=7460 GN=LOC552756 PE=3 SV=1 | 38.7 | 15 | 3 | 3 | 3 |
| Venom serine protease 34 | ✔ | A0A087ZYX8 | Venom serine protease 34 OS=Apis mellifera OX=7460 GN=SP28 PE=2 SV=1 | 44.6 | 15 | 5 | 19 | 5 |
| Major royal jelly protein 5 | ✔ | O97432 | Major royal jelly protein 5 OS=Apis mellifera OX=7460 GN=MRJP5 PE=2 SV=1 | 70.2 | 14 | 7 | 13 | 3 |
| Uncharacterized protein |  | A0A088AKU8 | Uncharacterized protein OS=Apis mellifera OX=7460 PE=3 SV=1 | 74.6 | 14 | 6 | 6 | 6 |
| Uncharacterized protein |  | A0A088AF62 | Uncharacterized protein OS=Apis mellifera OX=7460 GN=LOC725661 PE=4 SV=1 | 44.9 | 13 | 7 | 17 | 7 |
| Uncharacterized protein |  | A0A088ARD8 | Uncharacterized protein OS=Apis mellifera OX=7460 PE=3 SV=1 | 111 | 13 | 11 | 14 | 11 |
| Peptidase S1 domain-containing protein |  | A0A087ZRI9 | Peptidase S1 domain-containing protein OS=Apis mellifera OX=7460 PE=3 SV=1 | 36.9 | 13 | 3 | 4 | 3 |
| Uncharacterized protein |  | A0A088AC97 | Uncharacterized protein OS=Apis mellifera OX=7460 PE=4 SV=1 | 78.5 | 13 | 6 | 6 | 6 |
| Aldose 1-epimerase |  | A0A087ZNJ7 | Aldose 1-epimerase OS=Apis mellifera OX=7460 GN=LOC552086 PE=3 SV=1 | 42.2 | 12 | 3 | 10 | 3 |
| Uncharacterized protein |  | A0A087ZY30 | Uncharacterized protein OS=Apis mellifera OX=7460 PE=4 SV=1 | 23 | 12 | 2 | 8 | 2 |
| Uncharacterized protein |  | A0A087ZX58 | Uncharacterized protein OS=Apis mellifera OX=7460 GN=LOC551273 PE=4 SV=1 | 56.4 | 12 | 6 | 16 | 6 |
| Venom serine carboxypeptidase | ✔ | C9WMM5 | Venom serine carboxypeptidase OS=Apis mellifera OX=7460 PE=2 SV=1 | 53.7 | 12 | 4 | 6 | 4 |
| Uncharacterized protein |  | A0A087ZRA1 | Uncharacterized protein OS=Apis mellifera OX=7460 PE=3 SV=1 | 43 | 12 | 4 | 5 | 4 |
| Uncharacterized protein |  | A0A088AMA7 | Uncharacterized protein OS=Apis mellifera OX=7460 GN=LOC410539 PE=3 SV=1 | 65.8 | 11 | 5 | 6 | 5 |
| Uncharacterized protein |  | A0A088AQ33 | Uncharacterized protein OS=Apis mellifera OX=7460 PE=4 SV=1 | 51.1 | 11 | 3 | 5 | 3 |
| Beta-galactosidase |  | A0A088A3V9 | Beta-galactosidase OS=Apis mellifera OX=7460 GN=LOC725756 PE=3 SV=1 | 73 | 10 | 5 | 9 | 5 |
| Uncharacterized protein |  | A0A087ZXY2 | Uncharacterized protein OS=Apis mellifera OX=7460 GN=LOC413256 PE=4 SV=1 | 77.5 | 9 | 5 | 11 | 5 |
| HATPase_c domain-containing protein |  | H9KUJ6 | HATPase_c domain-containing protein OS=Apis mellifera OX=7460 GN=LOC412150 PE=3 SV=1 | 92.1 | 9 | 6 | 7 | 6 |
| Uncharacterized protein |  | A0A088AL93 | Uncharacterized protein OS=Apis mellifera OX=7460 PE=4 SV=1 | 61.5 | 8 | 2 | 2 | 2 |
| Alpha-glucosidase | ✔ | A0A087ZWK4 | Alpha-glucosidase OS=Apis mellifera OX=7460 PE=4 SV=1 | 67.5 | 8 | 4 | 7 | 4 |
| GMC_OxRdtase_N domain-containing protein |  | A0A088A031 | GMC_OxRdtase_N domain-containing protein OS=Apis mellifera OX=7460 PE=4 SV=1 | 67.8 | 8 | 4 | 4 | 4 |
| Uncharacterized protein |  | A0A087ZRN5 | Uncharacterized protein OS=Apis mellifera OX=7460 PE=4 SV=1 | 165 | 7 | 7 | 14 | 7 |
| Major royal jelly protein 3 | ✔ | Q3L632 | Major royal jelly protein 3 OS=Apis mellifera carnica OX=88217 GN=mrjp3 PE=4 SV=1 | 65.7 | 7 | 4 | 8 | 4 |
| Glyco_tran_10_N domain-containing protein |  | A0A087ZVV0 | Glyco_tran_10_N domain-containing protein OS=Apis mellifera OX=7460 GN=100578168 PE=3 SV=1 | 56.2 | 7 | 3 | 5 | 3 |
| Glucosylceramidase |  | A0A088APM4 | Glucosylceramidase OS=Apis mellifera OX=7460 GN=LOC409708 PE=3 SV=1 | 59.3 | 7 | 2 | 2 | 2 |
| Peptidase S1 domain-containing protein |  | A0A088AFB7 | Peptidase S1 domain-containing protein OS=Apis mellifera OX=7460 GN=SP13 PE=3 SV=1 | 50.2 | 6 | 2 | 2 | 2 |
| GMC_OxRdtase_N domain-containing protein |  | A0A087ZVX2 | GMC_OxRdtase_N domain-containing protein OS=Apis mellifera OX=7460 PE=4 SV=1 | 73 | 6 | 3 | 3 | 3 |
| Uncharacterized protein |  | A0A088A1A1 | Uncharacterized protein OS=Apis mellifera OX=7460 PE=4 SV=1 | 112 | 5 | 4 | 4 | 4 |
| Uncharacterized protein |  | A0A088A5N6 | Uncharacterized protein OS=Apis mellifera OX=7460 PE=4 SV=1 | 177 | 5 | 6 | 7 | 6 |
| Uncharacterized protein |  | A0A088AFD1 | Uncharacterized protein OS=Apis mellifera OX=7460 PE=4 SV=1 | 64 | 3 | 2 | 2 | 2 |
| Vitellogenin | ✔ | A0A088ADL8 | Vitellogenin OS=Apis mellifera OX=7460 PE=4 SV=1 | 201 | 3 | 4 | 4 | 4 |
| Aa_trans domain-containing protein |  | A0A088AP93 | Aa_trans domain-containing protein OS=Apis mellifera OX=7460 PE=4 SV=1 | 105 | 2 | 2 | 3 | 2 |
