# Supplementary material for: Factors driving the compositional diversity of Apis mellifera bee venom from a Corymbia calophylla (marri) ecosystem, Southwestern Australia
Source: PLoS One. 2021 Jun 30;16(6):e0253838. doi: 10.1371/journal.pone.0253838 (PMC8244862; doi:10.1371/journal.pone.0253838)
Supplement: S3 Table — A complementary list to Fig 3, presenting higher abundant proteins represented by the combined peptide match score (Sum PEP Score) and number of peptide spectrum matches (PSMs). MW: Molecular weight of intact protein; kDa (kilodalton). (DOCX) [file pone.0253838.s005.docx]

**S3 Table.**

| **Protein Accession** | **Description** | **Sum PEP Score** | **Coverage [%]** | **# Peptides** | **# PSMs** | **# Unique Peptides** | **# AAs** | **MW [kDa]** |
| --- | --- | --- | --- | --- | --- | --- | --- | --- |
| P00630 | Phospholipase A2 OS=Apis mellifera OX=7460 PE=1 SV=3 | 707.458 | 77 | 69 | 14735 | 69 | 167 | 19 |
| P01501 | Melittin OS=Apis mellifera OX=7460 GN=MELT PE=1 SV=1 | 561.933 | 69 | 41 | 14533 | 23 | 70 | 7.6 |
| Q08169 | Hyaluronidase OS=Apis mellifera OX=7460 PE=1 SV=1 | 443.734 | 62 | 40 | 4474 | 1 | 382 | 44.2 |
| A0A088AMF4 | Hyaluronidase OS=Apis mellifera OX=7460 PE=3 SV=1 | 439.496 | 62 | 40 | 4461 | 1 | 382 | 44.2 |
| A0A088AG18 | Uncharacterized protein OS=Apis mellifera OX=7460 GN=Sap-r PE=4 SV=1 | 326.875 | 45 | 43 | 2325 | 43 | 887 | 100 |
| A0A088AUL3 | PDGF_2 domain-containing protein OS=Apis mellifera OX=7460 GN=Pvf1 PE=3 SV=1 | 313.956 | 56 | 27 | 1949 | 27 | 318 | 35.9 |
| P0DPR9 | Melittin-N OS=Apis cerana OX=7461 GN=MELT PE=1 SV=1 | 300.437 | 67 | 22 | 2872 | 4 | 70 | 7.6 |
| Q4ZJX1 | Major royal jelly protein 9 OS=Apis mellifera OX=7460 GN=MRJP9 PE=2 SV=1 | 214.45 | 58 | 23 | 1468 | 23 | 423 | 48.7 |
| Q8MQS8 | Venom serine protease 34 OS=Apis mellifera OX=7460 PE=2 SV=1 | 213.409 | 55 | 20 | 606 | 20 | 405 | 45.5 |
| A0A087ZRC7 | Venom acid phosphatase Acph-1 OS=Apis mellifera OX=7460 GN=Acph-1 PE=3 SV=1 | 186.959 | 51 | 21 | 1195 | 21 | 401 | 46.8 |
| O18330 | Major royal jelly protein 1 OS=Apis mellifera OX=7460 GN=MRJP1 PE=1 SV=1 | 149.295 | 52 | 18 | 133 | 17 | 432 | 48.9 |
| A0A088A1D0 | Venom dipeptidyl peptidase 4 OS=Apis mellifera OX=7460 PE=3 SV=1 | 147.18 | 32 | 19 | 640 | 19 | 779 | 88.4 |
| A0A087ZZK8 | PA2c domain-containing protein OS=Apis mellifera OX=7460 GN=LOC724436 PE=3 SV=1 | 134.396 | 55 | 15 | 1519 | 15 | 174 | 20.5 |
| P83563 | Allergen Api m 6.03 / Api m 6.04 OS=Apis mellifera OX=7460 PE=1 SV=2 | 129.292 | 51 | 17 | 3255 | 17 | 92 | 9.8 |
| A0A087ZQ29 | Uncharacterized protein OS=Apis mellifera OX=7460 PE=4 SV=1 | 123.758 | 37 | 17 | 558 | 17 | 519 | 61.4 |
| O77061 | Major royal jelly protein 2 OS=Apis mellifera OX=7460 GN=MRJP2 PE=1 SV=1 | 113.497 | 46 | 17 | 232 | 1 | 452 | 51 |
| A0A1Q1N6G0 | Major royal jelly protein 2 OS=Apis mellifera carnica OX=88217 GN=MRJP2 PE=4 SV=1 | 111.681 | 46 | 17 | 229 | 1 | 452 | 51.1 |
| H9KQJ7 | Omega-conotoxin-like protein 1 OS=Apis mellifera OX=7460 PE=2 SV=1 | 108 | 43 | 7 | 531 | 7 | 74 | 8.3 |
| P01500 | Apamin OS=Apis mellifera OX=7460 PE=1 SV=2 | 103.583 | 54 | 10 | 636 | 10 | 46 | 5.2 |
| I1VC86 | Phospholipase A2 OS=Apis mellifera OX=7460 PE=2 SV=1 | 96.627 | 41 | 8 | 5596 | 8 | 167 | 19 |
| Q5EF78 | Icarapin OS=Apis mellifera carnica OX=88217 PE=1 SV=2 | 90.344 | 34 | 11 | 1638 | 2 | 223 | 24.8 |
| A0A096XH35 | Icarapin variant 2 (Fragment) OS=Apis mellifera carnica OX=88217 GN=icarapin PE=2 SV=1 | 87.11 | 36 | 11 | 1637 | 2 | 200 | 22.4 |
| A0A088A5R9 | Peptidyl-prolyl cis-trans isomerase OS=Apis mellifera OX=7460 PE=3 SV=1 | 80.078 | 52 | 9 | 516 | 9 | 184 | 20.1 |
| A0A087ZN44 | Carboxypeptidase OS=Apis mellifera OX=7460 PE=3 SV=1 | 76.057 | 31 | 7 | 241 | 7 | 409 | 46.8 |
| B2D0J5 | Venom carboxylesterase-6 OS=Apis mellifera OX=7460 PE=2 SV=1 | 75.788 | 21 | 11 | 652 | 11 | 557 | 63.6 |
| Q3L632 | Major royal jelly protein 3 OS=Apis mellifera carnica OX=88217 GN=mrjp3 PE=4 SV=1 | 75.777 | 26 | 12 | 213 | 11 | 579 | 65.7 |
| A0A087ZN65 | Uncharacterized protein OS=Apis mellifera OX=7460 PE=3 SV=1 | 73.046 | 54 | 8 | 373 | 8 | 157 | 17.9 |
| A0A087ZRA1 | Uncharacterized protein OS=Apis mellifera OX=7460 PE=3 SV=1 | 72.867 | 32 | 11 | 17 | 11 | 388 | 43 |
| O97432 | Major royal jelly protein 5 OS=Apis mellifera OX=7460 GN=MRJP5 PE=2 SV=1 | 67.128 | 23 | 11 | 77 | 3 | 598 | 70.2 |
| A0A088AU22 | Uncharacterized protein OS=Apis mellifera OX=7460 PE=4 SV=1 | 65.315 | 29 | 11 | 115 | 2 | 419 | 47.5 |
| A0A088A015 | Uncharacterized protein OS=Apis mellifera OX=7460 GN=LOC408851 PE=3 SV=1 | 64.388 | 17 | 12 | 295 | 12 | 881 | 100.3 |
| A0A087ZPM2 | Mast cell degranulating peptide OS=Apis mellifera OX=7460 GN=Mcdp PE=4 SV=1 | 56.696 | 48 | 9 | 1075 | 9 | 50 | 5.8 |
| Q6TGR0 | Major royal jelly protein 8 OS=Apis mellifera OX=7460 GN=Mrjp8 PE=2 SV=1 | 56.403 | 29 | 11 | 341 | 11 | 416 | 46.9 |
| A0A087ZWK4 | Alpha-glucosidase OS=Apis mellifera OX=7460 PE=4 SV=1 | 53.888 | 25 | 11 | 58 | 11 | 583 | 67.5 |
| A0A088AL69 | Uncharacterized protein OS=Apis mellifera OX=7460 PE=4 SV=1 | 53.221 | 46 | 6 | 272 | 6 | 140 | 16.4 |
| H9K869 | Uncharacterized protein OS=Apis mellifera OX=7460 PE=4 SV=1 | 47.462 | 50 | 8 | 702 | 8 | 96 | 11.4 |
| A0A088AUP4 | Uncharacterized protein OS=Apis mellifera OX=7460 GN=LOC724341 PE=4 SV=1 | 44.813 | 37 | 8 | 187 | 8 | 243 | 27.5 |
| A0A088AMB8 | ATP synthase subunit beta OS=Apis mellifera OX=7460 GN=Atp5b PE=3 SV=1 | 41.734 | 21 | 8 | 8 | 8 | 516 | 55.1 |
| A0A088AL68 | Secapin OS=Apis mellifera OX=7460 GN=LOC406145 PE=4 SV=1 | 41.106 | 36 | 4 | 4161 | 4 | 77 | 8.7 |
| A0A087ZPM1 | Tertiapin OS=Apis mellifera OX=7460 PE=4 SV=1 | 37.491 | 36 | 6 | 1308 | 6 | 53 | 6.1 |
| A0A087ZYN8 | Uncharacterized protein OS=Apis mellifera OX=7460 PE=4 SV=1 | 36.964 | 28 | 6 | 537 | 6 | 99 | 11.2 |
| A0A088A2Y0 | TIL domain-containing protein OS=Apis mellifera OX=7460 GN=100576444 PE=4 SV=1 | 34.347 | 48 | 3 | 62 | 3 | 82 | 9 |
| A0A088AP79 | Uncharacterized protein OS=Apis mellifera OX=7460 GN=LOC409790 PE=4 SV=1 | 29.358 | 17 | 3 | 179 | 3 | 211 | 24.3 |
| A0A087ZTN5 | Sep15_SelM domain-containing protein OS=Apis mellifera OX=7460 GN=LOC410663 PE=4 SV=1 | 28.156 | 32 | 4 | 136 | 4 | 155 | 17.8 |
| A0A088AQ33 | Uncharacterized protein OS=Apis mellifera OX=7460 PE=4 SV=1 | 25.951 | 12 | 3 | 11 | 3 | 483 | 51.1 |
| A0A088ACF4 | Uncharacterized protein OS=Apis mellifera OX=7460 PE=3 SV=1 | 25.099 | 8 | 5 | 24 | 5 | 655 | 72.4 |
| A0A088AF77 | Chymotrypsin inhibitor OS=Apis mellifera OX=7460 GN=Amci PE=4 SV=1 | 24.218 | 55 | 4 | 222 | 4 | 76 | 8.1 |
| A0A088AUD4 | Uncharacterized protein OS=Apis mellifera OX=7460 PE=3 SV=1 | 23.553 | 14 | 5 | 5 | 4 | 351 | 40.1 |
| A0A087ZXG5 | C1q-like venom protein OS=Apis mellifera OX=7460 PE=4 SV=1 | 23.35 | 27 | 6 | 306 | 6 | 177 | 18.7 |
| A0A087ZU97 | Uncharacterized protein OS=Apis mellifera OX=7460 GN=100578816 PE=4 SV=1 | 20.595 | 54 | 4 | 94 | 4 | 100 | 10.9 |
| A0A088AC16 | Uncharacterized protein OS=Apis mellifera OX=7460 GN=LOC408608 PE=4 SV=1 | 19.217 | 10 | 1 | 2 | 1 | 181 | 19.4 |
| Q1W633 | OBP21 OS=Apis mellifera OX=7460 GN=Obp21 PE=2 SV=1 | 19.038 | 33 | 4 | 4 | 4 | 135 | 15.2 |
| A0A088ABP1 | Uncharacterized protein OS=Apis mellifera OX=7460 PE=4 SV=1 | 18.722 | 36 | 5 | 6 | 5 | 173 | 17.6 |
| A0A087ZYW8 | Uncharacterized protein OS=Apis mellifera OX=7460 PE=4 SV=1 | 18.566 | 13 | 3 | 46 | 3 | 304 | 34.3 |
| A0A088AU27 | Uncharacterized protein OS=Apis mellifera OX=7460 PE=4 SV=1 | 18.406 | 8 | 4 | 43 | 2 | 445 | 50.8 |
| A0A088AMK2 | Glyco_18 domain-containing protein OS=Apis mellifera OX=7460 PE=3 SV=1 | 16.776 | 9 | 3 | 9 | 3 | 449 | 50.1 |
| A0A088AEY5 | Peptidase S1 domain-containing protein OS=Apis mellifera OX=7460 PE=3 SV=1 | 14.828 | 8 | 2 | 149 | 2 | 260 | 29.5 |
| P56587 | Tertiapin OS=Apis mellifera OX=7460 PE=1 SV=1 | 14.515 | 76 | 2 | 710 | 2 | 21 | 2.5 |
| A0A088A9D8 | Uncharacterized protein OS=Apis mellifera OX=7460 GN=LOC408414 PE=3 SV=1 | 14.38 | 11 | 3 | 3 | 2 | 284 | 32.1 |
| B9UKD2 | Defensin OS=Apis cerana cerana OX=94128 GN=Def PE=2 SV=1 | 14.294 | 27 | 2 | 19 | 2 | 95 | 10.7 |
| A0A087ZTZ3 | Uncharacterized protein OS=Apis mellifera OX=7460 GN=100577847 PE=4 SV=1 | 13.909 | 7 | 2 | 38 | 2 | 376 | 42.2 |
| A0A087ZRB6 | Uncharacterized protein OS=Apis mellifera OX=7460 GN=LOC725215 PE=3 SV=1 | 13.804 | 12 | 3 | 21 | 3 | 361 | 42.6 |
| A0A087ZTA5 | ML domain-containing protein OS=Apis mellifera OX=7460 GN=LOC724386 PE=4 SV=1 | 13.771 | 25 | 2 | 2 | 2 | 148 | 16.1 |
| A0A088AJE9 | J domain-containing protein OS=Apis mellifera OX=7460 PE=4 SV=1 | 13.612 | 8 | 2 | 41 | 2 | 358 | 40.4 |
| Q1W640 | OBP14 OS=Apis mellifera OX=7460 PE=1 SV=1 | 12.522 | 24 | 2 | 41 | 2 | 135 | 15.2 |
| A0A088AF71 | TIL domain-containing protein OS=Apis mellifera OX=7460 GN=LOC725202 PE=4 SV=1 | 12.266 | 20 | 1 | 3 | 1 | 74 | 8.1 |
| A0A088A031 | GMC_OxRdtase_N domain-containing protein OS=Apis mellifera OX=7460 PE=4 SV=1 | 11.895 | 5 | 3 | 8 | 3 | 615 | 67.8 |
| A0A088A045 | ML domain-containing protein OS=Apis mellifera OX=7460 PE=4 SV=1 | 11.285 | 21 | 2 | 54 | 2 | 154 | 16.8 |
| Q06601 | Apidaecins type 14 OS=Apis mellifera OX=7460 GN=APID14 PE=1 SV=1 | 10.144 | 30 | 1 | 22 | 1 | 168 | 19.4 |
| A0A088AST9 | Enolase OS=Apis mellifera OX=7460 PE=4 SV=1 | 10.1 | 5 | 1 | 1 | 1 | 366 | 39.8 |
| A0A087ZSL8 | Uncharacterized protein OS=Apis mellifera OX=7460 PE=4 SV=1 | 9.965 | 7 | 2 | 62 | 2 | 289 | 32.2 |
| Q10416 | Hymenoptaecin OS=Apis mellifera OX=7460 PE=2 SV=1 | 9.673 | 31 | 4 | 25 | 4 | 129 | 14.5 |
| A0A088AHC8 | Glyceraldehyde-3-phosphate dehydrogenase OS=Apis mellifera OX=7460 GN=Gapdh PE=2 SV=1 | 9.179 | 8 | 2 | 2 | 2 | 333 | 35.9 |
| Q8N0N7 | Alpha-amylase OS=Apis mellifera mellifera OX=44477 PE=3 SV=1 | 9.153 | 4 | 1 | 2 | 1 | 493 | 56 |
| A0A088APM4 | Glucosylceramidase OS=Apis mellifera OX=7460 GN=LOC409708 PE=3 SV=1 | 9.022 | 7 | 2 | 5 | 2 | 522 | 59.3 |
| A0A088AF74 | TIL domain-containing protein OS=Apis mellifera OX=7460 GN=LOC725114 PE=4 SV=1 | 9.012 | 21 | 2 | 2 | 2 | 90 | 10.1 |
| A0A088A1A1 | Uncharacterized protein OS=Apis mellifera OX=7460 PE=4 SV=1 | 8.642 | 2 | 2 | 2 | 2 | 1010 | 112.1 |
| A0A088AF62 | Uncharacterized protein OS=Apis mellifera OX=7460 GN=LOC725661 PE=4 SV=1 | 8.377 | 6 | 2 | 8 | 2 | 390 | 44.9 |
| A0A088APL1 | Chitin-binding type-2 domain-containing protein OS=Apis mellifera OX=7460 GN=Cht5 PE=3 SV=1 | 8.013 | 4 | 2 | 2 | 2 | 605 | 69 |
| A0A088AVY1 | Ubiquitin-like domain-containing protein OS=Apis mellifera OX=7460 GN=RpS27A PE=4 SV=1 | 8.008 | 12 | 2 | 8 | 2 | 156 | 17.9 |
| A0A088A4U8 | Uncharacterized protein OS=Apis mellifera OX=7460 PE=4 SV=1 | 6.547 | 6 | 1 | 2 | 1 | 185 | 19.7 |
| A0A088ADT0 | Uncharacterized protein OS=Apis mellifera OX=7460 PE=4 SV=1 | 6.151 | 5 | 1 | 40 | 1 | 237 | 27.8 |
| A0A088AIU0 | Chitin-binding type-2 domain-containing protein OS=Apis mellifera OX=7460 PE=4 SV=1 | 5.648 | 6 | 1 | 1 | 1 | 178 | 20.5 |
| A0A088A0F9 | Uncharacterized protein OS=Apis mellifera OX=7460 GN=LOC726118 PE=4 SV=1 | 5.017 | 15 | 1 | 50 | 1 | 78 | 9.5 |
| A0A088A5S0 | GPI inositol-deacylase OS=Apis mellifera OX=7460 PE=3 SV=1 | 4.578 | 1 | 1 | 480 | 1 | 847 | 96.9 |
| A0A088AQD8 | Uncharacterized protein OS=Apis mellifera OX=7460 GN=LOC724644 PE=4 SV=1 | 4.295 | 7 | 1 | 9 | 1 | 134 | 15.4 |
| A0A088AM30 | Uncharacterized protein OS=Apis mellifera OX=7460 GN=ATPsyn-Cf6 PE=4 SV=1 | 4.084 | 11 | 1 | 1 | 1 | 95 | 11 |
| A0A087ZUK9 | Malate dehydrogenase OS=Apis mellifera OX=7460 GN=LOC408950 PE=3 SV=1 | 4.023 | 3 | 1 | 1 | 1 | 338 | 35.8 |
| A0A088AU21 | Uncharacterized protein OS=Apis mellifera OX=7460 PE=4 SV=1 | 3.78 | 4 | 2 | 8 | 1 | 437 | 49.8 |
| A0A088ARX3 | Uncharacterized protein OS=Apis mellifera OX=7460 PE=4 SV=1 | 3.414 | 13 | 1 | 10 | 1 | 159 | 19.1 |
| A0A088A216 | Uncharacterized protein OS=Apis mellifera OX=7460 PE=4 SV=1 | 2.849 | 1 | 1 | 66 | 1 | 1315 | 150 |
| A0A087ENM7 | Uncharacterized protein OS=Lactobacillus kunkeei OX=148814 GN=JI66_05660 PE=4 SV=1 | 2.802 | 2 | 1 | 1 | 1 | 419 | 48.3 |
| A0A087ZX58 | Uncharacterized protein OS=Apis mellifera OX=7460 GN=LOC551273 PE=4 SV=1 | 2.751 | 2 | 1 | 1 | 1 | 492 | 56.4 |
| V5RL49 | Uncharacterized protein OS=Spiroplasma apis B31 OX=1276258 GN=SAPIS_v1c06750 PE=4 SV=1 | 2.707 | 15 | 1 | 2 | 1 | 257 | 30 |
| V5RJA1 | Histidine triad protein OS=Spiroplasma apis B31 OX=1276258 GN=hit PE=4 SV=1 | 2.689 | 17 | 1 | 3 | 1 | 137 | 15.7 |
| A0A087ZND8 | Uncharacterized protein OS=Apis mellifera OX=7460 GN=Mlc2 PE=4 SV=1 | 2.544 | 4 | 1 | 1 | 1 | 213 | 23.5 |
| A0A088ABE7 | Odorant receptor OS=Apis mellifera OX=7460 PE=3 SV=1 | 2.525 | 3 | 1 | 1 | 1 | 395 | 46.3 |
| A0A3S8UTR4 | Uncharacterized protein OS=Apis mellifera associated microvirus 56 OX=2494787 PE=4 SV=1 | 2.325 | 12 | 1 | 1 | 1 | 130 | 15.1 |
| I7KJQ1 | Troponin C type IIIa OS=Apis mellifera OX=7460 GN=TpnCIIIa PE=4 SV=1 | 2.292 | 6 | 1 | 1 | 1 | 153 | 17.3 |
| V5RIY8 | PTS system cellobiose-specific IIC component OS=Spiroplasma apis B31 OX=1276258 GN=celB1 PE=4 SV=1 | 2.291 | 1 | 1 | 94 | 1 | 729 | 82 |
| V5RJ10 | Arginine--tRNA ligase OS=Spiroplasma apis B31 OX=1276258 GN=argS PE=3 SV=1 | 2.26 | 1 | 1 | 4 | 1 | 553 | 63 |
| A0A088A932 | Uncharacterized protein OS=Apis mellifera OX=7460 PE=3 SV=1 | 2.243 | 0 | 1 | 20 | 1 | 2118 | 239.7 |
| A0A087ZSJ1 | SGL domain-containing protein OS=Apis mellifera OX=7460 PE=4 SV=1 | 1.843 | 9 | 1 | 1 | 1 | 94 | 10.2 |
| A0A087ZZC1 | F5/8 type C domain-containing protein OS=Apis mellifera OX=7460 GN=LOC411212 PE=4 SV=1 | 1.097 | 1 | 1 | 16 | 1 | 632 | 72.2 |
| B0LUE8 | Apolipophorin-III-like protein OS=Apis mellifera OX=7460 GN=A4 PE=2 SV=1 | 1.038 | 4 | 1 | 1 | 1 | 193 | 21.3 |
