# Supplementary material for: Factors driving the compositional diversity of Apis mellifera bee venom from a Corymbia calophylla (marri) ecosystem, Southwestern Australia
Source: PLoS One. 2021 Jun 30;16(6):e0253838. doi: 10.1371/journal.pone.0253838 (PMC8244862; doi:10.1371/journal.pone.0253838)
Supplement: S4 Table — Bee venom weights and information on ecological and biological variables are presented (Temperature, Humidity, Nutritional supply, Historical hives’ movement, Behavioural Flowering index, Nectar volume). Behavioural response: active (>80 bees reacting to the stimulating device); docile (<80 bees reacting to the stimulating device); Nutritional supply: PSS: unspecified pollen plus soy flour plus sugar syrup; MP: marri pollen; N: None; Flowering index (MFI; according to Campbell and Fearns, 2018). (DOCX) [file pone.0253838.s006.docx]

**S4 Table**

| **Date** | **Number** | **Site** | **Temperature (°C)** | **Humidity %** | **Weight (g)** | **Hives' number** | **Behavioural response** | **Nutritional supply** | **Historical hives' movement** | **Flowering index** | **Nectar volume (µl)** |
| --- | --- | --- | --- | --- | --- | --- | --- | --- | --- | --- | --- |
| 31/01/2020 | 1 | Hovea | 20.7 | 36 | 0.148 | 1 | active | PSS | S | 6.65 | 1.22 |
| 31/01/2020 | 2 | Hovea | 20.7 | 36 | 0.096 | 2 | active | PSS | S | 6.65 | 1.22 |
| 31/01/2020 | 3 | Hovea | 20.7 | 36 | 0.101 | 3 | active | PSS | S | 6.65 | 1.22 |
| 31/01/2020 | 4 | Hovea | 20.7 | 36 | 0.117 | 4 | active | PSS | S | 6.65 | 1.22 |
| 31/01/2020 | 5 | Hovea | 20.7 | 36 | 0.080 | 5 | active | PSS | S | 6.65 | 1.22 |
| 10/2/2020 | 6 | Chidlow | 20.7 | 35 | 0.057 | 1 | active | MP | MS | 4.47 | 1.60 |
| 10/2/2020 | 7 | Chidlow | 20.7 | 35 | 0.097 | 2 | active | MP | MS | 4.47 | 1.60 |
| 10/2/2020 | 8 | Chidlow | 20.7 | 35 | 0.062 | 3 | active | MP | MS | 4.47 | 1.60 |
| 10/2/2020 | 9 | Chidlow | 20.7 | 35 | 0.066 | 4 | active | MP | MS | 4.47 | 1.60 |
| 10/2/2020 | 10 | Chidlow | 20.7 | 35 | 0.082 | 5 | active | MP | MS | 4.47 | 1.60 |
| 12/2/2020 | 11 | Harvey | 26 | 53 | 0.039 | 1 | active | N | M | 5.2 | 3.22 |
| 12/2/2020 | 12 | Harvey | 26 | 53 | 0.029 | 2 | active | N | M | 5.2 | 3.22 |
| 12/2/2020 | 13 | Harvey | 26 | 53 | 0.065 | 3 | active | N | M | 5.2 | 3.22 |
| 12/2/2020 | 14 | Harvey | 26 | 53 | 0.076 | 4 | active | N | M | 5.2 | 3.22 |
| 12/2/2020 | 15 | Harvey | 26 | 53 | 0.118 | 5 | active | N | M | 5.2 | 3.22 |
| 15/02/2020 | 16 | Chittering | 26.5 | 58 | 0.084 | 1 | active | N | M | 5.16 | 11.67 |
| 15/02/2020 | 17 | Chittering | 26.5 | 58 | 0.103 | 2 | active | N | M | 5.16 | 11.67 |
| 15/02/2020 | 18 | Chittering | 26.5 | 58 | 0.084 | 3 | active | N | M | 5.16 | 11.67 |
| 15/02/2020 | 19 | Chittering | 26.5 | 58 | 0.021 | 4 | active | N | M | 5.16 | 11.67 |
| 15/02/2020 | 20 | Chittering | 26.5 | 58 | 0.047 | 5 | active | N | M | 5.16 | 11.67 |
| 22/02/2020 | 21 | Byford | 27 | 58 | 0.012 | 1 | docile | N | M | 6.05 | 13.46 |
| 22/02/2020 | 22 | Byford | 27 | 58 | 0.012 | 2 | docile | N | M | 6.05 | 13.46 |
| 22/02/2020 | 23 | Byford | 27 | 58 | 0.037 | 3 | active | N | M | 6.05 | 13.46 |
| 22/02/2020 | 24 | Byford | 27 | 58 | 0.003 | 4 | docile | N | M | 6.05 | 13.46 |
| 22/02/2020 | 25 | Byford | 27 | 58 | 0.023 | 5 | docile | N | M | 6.05 | 13.46 |
| 25/02/2020 | 26 | Harvey | 26.3 | 54 | 0.039 | 1 | active | N | M | 3.85 | 4.28 |
| 25/02/2020 | 27 | Harvey | 26.3 | 54 | 0.112 | 2 | active | N | M | 3.85 | 4.28 |
| 25/02/2020 | 28 | Harvey | 26.3 | 54 | 0.035 | 3 | active | N | M | 3.85 | 4.28 |
| 25/02/2020 | 29 | Harvey | 26.3 | 54 | 0.096 | 4 | active | N | M | 3.85 | 4.28 |
| 25/02/2020 | 30 | Harvey | 26.3 | 54 | 0.111 | 5 | active | N | M | 3.85 | 4.28 |
| 28/02/2020 | 31 | Hovea | 22.4 | 71 | 0.030 | 1 | active | PSS | S | 5.58 | 4.50 |
| 28/02/2020 | 32 | Hovea | 22.4 | 71 | 0.058 | 2 | active | PSS | S | 5.58 | 4.50 |
| 28/02/2020 | 33 | Hovea | 22.4 | 71 | 0.032 | 3 | active | PSS | S | 5.58 | 4.50 |
| 28/02/2020 | 34 | Hovea | 22.4 | 71 | 0.079 | 4 | active | PSS | S | 5.58 | 4.50 |
| 28/02/2020 | 35 | Hovea | 22.4 | 71 | 0.019 | 5 | docile | PSS | S | 5.58 | 4.50 |
| 1/3/2020 | 36 | Chittering | 24.9 | 50 | 0.051 | 1 | active | N | M | 6 | 17.65 |
| 1/3/2020 | 37 | Chittering | 24.9 | 50 | 0.158 | 2 | active | N | M | 6 | 17.65 |
| 1/3/2020 | 38 | Chittering | 24.9 | 50 | 0.078 | 3 | active | N | M | 6 | 17.65 |
| 1/3/2020 | 39 | Chittering | 24.9 | 50 | 0.032 | 4 | active | N | M | 6 | 17.65 |
| 1/3/2020 | 40 | Chittering | 24.9 | 50 | 0.034 | 5 | active | N | M | 6 | 17.65 |
| 4/3/2020 | 41 | Byford | 30.7 | 21 | 0.004 | 1 | docile | N | M | 5.75 | 0.00 |
| 4/3/2020 | 42 | Byford | 30.7 | 21 | 0.015 | 2 | docile | N | M | 5.75 | 0.00 |
| 4/3/2020 | 43 | Byford | 30.7 | 21 | 0.030 | 3 | active | N | M | 5.75 | 0.00 |
| 4/3/2020 | 44 | Byford | 30.7 | 21 | 0.005 | 4 | docile | N | M | 5.75 | 0.00 |
| 4/3/2020 | 45 | Byford | 30.7 | 21 | 0.004 | 5 | docile | N | M | 5.75 | 0.00 |
| 5/3/2020 | 46 | Chidlow | 24.4 | 35 | 0.001 | 1 | docile | MP | MS | 6.49 | 0.60 |
| 5/3/2020 | 47 | Chidlow | 24.4 | 35 | 0.047 | 2 | active | MP | MS | 6.49 | 0.60 |
| 5/3/2020 | 48 | Chidlow | 24.4 | 35 | 0.016 | 3 | active | MP | MS | 6.49 | 0.60 |
| 5/3/2020 | 49 | Chidlow | 24.4 | 35 | 0.017 | 4 | docile | MP | MS | 6.49 | 0.60 |
| 5/3/2020 | 50 | Chidlow | 24.4 | 35 | 0.026 | 5 | active | MP | MS | 6.49 | 0.60 |
| 6/3/2020 | 51 | Hovea | 21.6 | 67 | 0.003 | 1 | docile | PSS | S | 5.2 | 1.83 |
| 6/3/2020 | 52 | Hovea | 21.6 | 67 | 0.009 | 2 | docile | PSS | S | 5.2 | 1.83 |
| 6/3/2020 | 53 | Hovea | 21.6 | 67 | 0.007 | 3 | docile | PSS | S | 5.2 | 1.83 |
| 6/3/2020 | 54 | Hovea | 21.6 | 67 | 0.019 | 4 | active | PSS | S | 5.2 | 1.83 |
| 6/3/2020 | 55 | Hovea | 21.6 | 67 | 0.003 | 5 | docile | S | S | 5.2 | 1.83 |
