# Supplementary material for: Factors driving the compositional diversity of Apis mellifera bee venom from a Corymbia calophylla (marri) ecosystem, Southwestern Australia
Source: PLoS One. 2021 Jun 30;16(6):e0253838. doi: 10.1371/journal.pone.0253838 (PMC8244862; doi:10.1371/journal.pone.0253838)
Supplement: S1 Appendix — (DOCX) [file pone.0253838.s007.docx]

**S1 Appendix**

**Background**

Bee venom samples were collected during the flowering season of *Corymbia calophylla* (marri). Despite observations that *C. calophylla* was the primary floral component at the time of sampling, a quantitative analysis was necessary to determine if marri pollen represented the principal food source for bees (*Apis mellifera*) during the bee venom sampling period.

**Methods**

Pollen was identified from 10 honey samples (Table S1) collected from hives at the study sites, and one trap pollen sample each from Harvey, Chittering and Byford. The samples were collected at the end of the flowering season of *C. calophylla.* Honey samples (20 mL) were diluted (1:10) with distilled H_2_O, heated and rinsed-centrifuged at 3,000 rpm for 3 minutes several times to remove honey and retain the pollen content as a residue. The pollen residue was sieved with a 120 µm nylon mesh to remove extraneous bee parts. Approximately 5 gm of trap pollen per sample was dispersed in warm distilled H_2_O and rinsed-centrifuged as above several times, and sieved. The honey pollen and trap pollen residues were then acetolysed as per Erdtman (1960), and mounted on glass microscope slides in a permanent plastic mounting medium. Pollen was examined under high magnification with an Olympus-BX 51 microscope with Olympus–DP71 camera (Olympus, Tokyo, Japan), and identified by comparison with acetolysed mounted pollen samples from herbarium specimens of Eucalypt species and other commonly co-flowering plant taxa.

**Results**

Pollen analysis (Table S1) showed that pollen in the honey samples were predominantly from marri (*C. calophylla*), ranging from 67.70% in Byford to 94.54% in Harvey. Other species of Myrtaceae present included jarrah (*Eucalyptus marginata*) with up to 7.50% at Chittering and blackbutt (*Eucalyptus patens*), ranging from 1.36% to 21.01% at Byford. Similarly, marri pollen comprised 74.23-94% of the trap pollen samples.

**Conclusions**

As expected, the major food plant for bees during the bee venom collection period was marri (*C. calophylla*), indicating that the bee diet relied primarily on this most abundant flowering source.

**References**

Erdtman G. The acetolysis method: a revised description. Svensk Botanisk Tidskrift 1960 54: 341–35
