# Supplementary material for: Factors driving the compositional diversity of Apis mellifera bee venom from a Corymbia calophylla (marri) ecosystem, Southwestern Australia
Source: PLoS One. 2021 Jun 30;16(6):e0253838. doi: 10.1371/journal.pone.0253838 (PMC8244862; doi:10.1371/journal.pone.0253838)
Supplement: Attachment — Submitted filename: Responses to reviewers.docx [file pone.0253838.s008.docx]

**Reviewer #1:** The manuscript is interesting and the integration of different approaches is appreciable. I have no major remarks but some minor issues. –

First of all the authors must revise the whole manuscript in terms of grammar and readability. Some sentences are quite odd or very hard to follow as they stand now. Please also check the typos in the presented figures - Figures are really too much and most of them could be grouped in composite boxes ore moved to the supplementary information. Figure 3 is completely awkward and impossible to read, whereas the PCA graphs seem quite unnecessary since no clear groups are visible.

>>We improved the readability of the whole manuscript and fixed typos in the presented figures. We replaced Figure 3 making it more legible and moved the unnecessary PCAs - Fig. 5 and 8 - into the Supplementary information. For complementing Figure 3 and avoiding overlap of multiple protein names, we created a novel ‘S2 Table’ that refers to the figure and presents the full list of proteins with relative names.

ABSTRACT

line 19: please, provide a brief context to the applicative relevance of BV

>>Provided.

Line 31-32: the sentence seems truncated. Please check.

>>We have clarified the sentence.

Lines 36-37: Unclear sentence. Please rewrite.

>>Rewritten.

Line 37: please, be clearer concerning the investigated behaviour.

>>Clarified.

INTRODUCTION: line 112: which is the relevance of this plant in the context of the study?

>>Included.

METHODS:

lines 135-136: this sentence should be placed in the introduction

>>Removed from the methods and moved in the introduction.

line 140: use the comma to delimit thousands.

>>Fixed.

line 144 fig.1: unclear sentence. Please rewrite

>>Rewritten.

DISCUSSION:

line 370: please be clear about the issue of "certification". Is certification currently adopted for BV commercialization? Which kind of certification procedure are currently required for this product? Is there any international standard for certification of BV?

>>Currently, there aren’t tests for quality assurance or certification program for BV. Targeted sentences have been implemented to contextualise this issue.

L54-58

LL386-387

LL532-533

lines 373-377: this part seems an abstract/introduction. I would suggest to remove it from this chapter.

>>Removed.

line 387: Did you measure the differences among colonies at the same site? Could this factor influence the site-specificity of BV yield and features?

>>Thanks the reviewer for the comment. This is surely an aspect that deserves to be investigated by further work. However, to spot this variation further samplings per site per flowering season might better reveal the trend. At this stage we set a macro-scale approach according to our study aims, testing BV compositional diversity driven by geographical variation among sites. To partly discuss this aspect, we have provided elucidations that are adherent to our current data.

L404-405

L482-483

CONCLUSION: Apart from the future perspectives, this chapter looks like more as an abstract rather than a conclusion section. I would suggest to rewrite it.

>>The conclusion has been revisited.

Line 480: remove the word "fresh". It is not appropriate.

>>Removed.

**Reviewer #2**: In this manuscript the authors studied the influence of several ecological, management and behavioural factors in bee venom weight and protein composition. Bee venom (BV) is produced by honeybees and has been widely studied and increasingly sought for its therapeutic features and pharmaceutical applications. Several studies have explored the biological factors influencing bee venom protein nature and amount, but little research have analyzed the role of ecological factors.

For this reason I think that this article is original, as based on an advanced proteomics technique combined with both ecological and biological information for data interpretation and prediction of causes behind BV variance. Authors provide an useful tool based on an extensive proteins’ assemblage of aid for establishing BV certification programs.

This tool would also address the growing demand for BV in clinical and therapeutic fields, with substantial benefit to both human health and the beekeeping industry/primary producers. The experimental plan is well organized, statistical analysis are appropriate and results are well argued.

However, I believe that merging the results and discussion into a single section (Results and Discussion) would improve the quality of the article.

Moreover, I suggest the following minor revision:

- Figure 3 is not legible: use acronyms instead of whole names of the proteins, so as to avoid overlapping writings; moreover change the color of the bubbles to make writings visible;

>>We replaced Figure 3 making it more legible.

- In figure 4 the legend is not necessary as the locations are shown in the base of the graph;

>>We removed the legend.

- Add the degree of significance in the boxplots in figures 4,7,9;

>>Added.

- In figures 5,6,8 check the percentages in axis 1 and 2 carefully. They are different from those reported in the main text;

>>Fixed in the main text.

- Lines 102-103: add the reference number for Ferreira et al (2010);

>>Added.

- Line 269: the authors writes “three target proteins”, but four proteins are listed between parentheses;

>>Corrected into ‘four’.

- Line 445: replace (Fig1) with (Fig 1).

>>Done.

- While revising your manuscript refer to the downloadable sample files to ensure that your submission meets the formatting requirements. References are not listed according to the guidelines of the Journal. Correct them.

>>Corrected.

- Please upload your figure files to the Preflight Analysis and Conversion Engine (PACE) digital diagnostic tool, http://pace.apexcovantage.com/. PACE helps ensure that figures meet PLOS requirements. To use PACE, you must first register as a user. Registration is free. Then, login and navigate to the UPLOAD tab, where you will find detailed instructions on how to use the tool.

>>Done.
